# Spirit of the Foreign Periodicals

**Published:** 1839

**Authors:** 


### Spirit of the Foreign Periodicals.

### Decline of Broussaism in France.

In a recent biographical memoir of the medical reformer of the nineteenth century, as he used to be called by his admirers, we find the following most judicious remarks, which our readers will observe are in strict accordance with the opinion which the Medico-Chirurgical Review has uniformly expressed on the doctrines of the Broussaian school.

* * * * * "Thus the pains he took upon all occasions to connect the existing symptoms of a disease with certain suffering organs, to ascend thereby from the effect to the cause, and to take as the *point de depart* a rational and experimental demonstration, perhaps also the desire and secret hope to arrive at length at something fixed and determinate in medicine, contributed to impart a great value to the opinion of Broussais. Every one was struck with the sagacity of his criticism, with the depth of his observation, with the subtlety of his reasonings, with the boldness and connexion of his assertions, and especially with the grandeur and importance of the results which were promised, almost announced, as infallible.

What added to these causes of success was the dangerous *prestige* of pathological simplification and therapeutic unity, which pervaded the Broussaian theory: many allowed themselves to be led away by this attraction. A doctrine professing to be founded on two bases, irritation and *ab*-irritation, inflammation and sub-inflammation—or, in other words, the convenient but false dogmatic principle of mere *excess* and *defect* as a cause of all morbid action, supported too by the imposing authority and admirable connexion of its formularies, by the vast multiplicity of its facts, and by the rigour of its conclusions, seemed, and indeed must have seemed, to be of an almost unassailable solidity. There appeared at first sight to be a cohesion at once so strict and so profound of the principles of this system, that a leading proof of its truth was drawn from this very fallacy. *Broussais* had a degree of boldness in laying down principles, and in drawing from phenomena the deductions which favoured his views, that all opposition was overthrown, and admiration extorted from his very opponents. It was thus that he contrived to give a more exact and neat idea of fever—that obscure phantom of every age—than had ever been announced before. By the aid of his method, the medical art seemed to be rendered altogether more simple, more methodical, and at the same time more easy and more rigorous; for the indication to be pursued was always there, always present under the eyes of the physician to guide and enlighten him.

Even the very use of those fascinating words, *progress, advancement of science, novelty of doctrine,* contributed not a little to throw a brilliancy around a theory like that of Broussais, especially at an epoch when the political passions were in some degree allied to the movements of scientific enquiry. The epithets of servile or liberal, advancing or retrograde, were most freely bandied about, according as a party admitted or rejected the doctrines of irritation and the localisation of fevers. *Broussais* understood well the art of drawing to himself the hasty, fickle, and enthusiastic temper of youth; and, having done this, he applied himself to attract the more thoughtful and reflective. The consummate address with which he well knew how to present to his followers, whatever was really true and useful in his doctrine, and to keep back whatever was doubtful, and thus to steer between what was strictly proved, and what was perhaps utterly incorrect, contributed not a little to captivate the minds of many of his followers.

We need not therefore be much surprised if, at the *apogee of physiologism*, this distinguished physician, intoxicated with the incense of popularity, and elated with his own thoughts, shewed a sort of hostile warring pride, erecting his own opinions as an absolute rule and standard, and summoning to his tribunal, examining, judging, and condemning all other doctrines. Hippocrates himself did not find favour with the reformer of the nineteenth century, and although he, like *Rasori*, has not called him the *father of error*, he looked upon him only as an old statue to be gazed upon by the multitude.

At the end of a few years however the halo of *physiologism* very sensibly failed; the puritans of this doctrine became less numerous and less zealous. It was perceived that *Broussais*, like all other reformers, was powerful in exposing error, but feeble in discovering truth. The objections, remarks, exceptions, and criticisms against the doctrine of irritation quickly increased. Medical men perceived that this morbid entity—so to speak—differed in different diseases, not in degree only, but in its very nature and essential characters. To regard therefore a disease as a mere quantitative deviation from the healthy standard of the physiological condition was to lose sight of the abnormal character of diseased action. It was evident that there is far from being an identity of action in all inflammations; it is only apparent; for very often they differ from each other more in speciality or essence, than in mere degree. Again, it is utterly impossible to establish the localisation of morbid affections in very numerous cases; the sympathy of diseased action preventing us from recognising distinctly the original seat, or *point de depart*, of the malady. *Broussais* and his followers too often confounded the effect with the primary cause of a disease, and at the same time forgot that there are other agencies or sources of disease besides organic lesions or changes of structure in a part. They entirely neglected the alteration of the fluids, henceforth indisputable, and especially that of the organ of organs—the blood; and they attached far too much importance to the *organism*, and too little to the *dyanism*, of the animal economy. Lastly, the therapeutic principles insisted upon by the new school of medicine were speedily discovered to be too often at issue with the actual results of practice; diseases got well under the use of means the very opposite to those recommended by the Broussaists; and the success of the *empirics*—the term is used in its hippocratic sense—was generally greater than that of the self-called *physiologists*.

It cannot be denied that the most potent cause of the decline of the Broussaian doctrines has been, that clinical experience has been found to be too much at variance with its promises and assurances. Physiologism is a plant which will not grow, unless it is constantly sprinkled with blood; the lancet is indispensable to its culture in all climates and seasons! Then too a most rigorous, implacable regimen was summoned to aid this means of cure.* Still the cures were certainly not numerous; irritation, that obstinate malicious being, continued to resist alike the assaults of bleeding and the blockade of starvation; and, if it did finally yield, the strength of the invalid was reduced to the lowest ebb, and his recovery was slow and imperfect.

In illustration of these remarks, we have only to remind our readers of the etiology and treatment of many stomach disorders inculcated by the Broussaian school. Every *gastralgia* was considered a *gastritis*, and was subjected to a most rigid course of depletory and antiphlogistic treatment; thus numerous

---

* An anecdote is told of Broussais' practice to the following effect. A patient, who had for some time submitted to the starving system, called upon him and said: "Your regimen, Doctor, has pulled down my strength to the last degree; it is killing me; and I am dying of hunger." Broussais, after looking at him for some time, answered, "Well, you carnivorous brute (bete carnassiere), I will satisfy you; you may have a spoonful of broth in a tumbler of water."

cases, which might have been speedily relieved by the use of bitter and tonic medicines, were trifled with, or rather aggravated by, a protracted employment of leeches, ptisans, and such-like means.

*Broussais* had no doubt been led, at least in some degree, into this error of practice by having had chiefly under his care, during his early professional career, none but vigorous and robust soldiers. A mode of treatment that is well adapted to military practice will not suit that of large towns. This is a truth which is well understood, and most attentively acted upon, by all experienced practitioners, both of Paris and London. The more experienced the practitioner, the less does he employ the lancet and other depletory means.

Well, after some years of most brilliant success, the great reformer began to find that he was gradually losing ground; his work ceased to sell; his journal was discontinued; his writings were subjected to a searching *examen*, and occasionally to the flagellation of sarcasm and ridicule; and at length, the doctrine of irritation, abandoned to itself, seemed to be counted among the errors of bye-gone times.

A convincing proof of this was, that *Broussais*, when appointed one of the professors of the faculty, failed to attract to his prelections the crowds of auditors that formerly hung upon his lips to receive the creed of instruction, only a few pupils attended; and even they seemed to be influenced more by curiosity than by admiration. The necrology of physiologism was visibly written in this desertion. It was at this period of his career, when his repute as a physician was so rapidly declining, that he stepped forward as a promulgator of phrenology, displaying the same talents as a vehement and impassioned declaimer, and as a subtle and attractive reasoner, as had made him, at a former period of his career, *the* lecturer of the day.

All his former prepossessions on subjects more strictly medical, tended to engage Broussais in the advocacy of Phrenology. He associated every manifestation of life, whether in the healthy or diseased state, with certain changes in certain parts or structures of the body; in other words, he was strictly an organologist, alike in his mental and in his physical philosophy.

It was to the following-out of this principle too rigidly that all his errors, both as a medical man and as a metaphysician, may be traced. He could not conceive, and therefore he would not admit, that any phenomenon in a living body could possibly be manifested without a specific and organic action in some particular part of the body. It was thus that, to his mind, the existence of those symptoms, to which we give the appellation of fever, suggested the inevitable existence of a local lesion in some organ or another of the body,—forgetting all the while that the body may be suffering seriously, and at every point of its frame, without a necessary lesion of any particular texture or structure. The same is probably true of the mind. We do not dispute that there is a strict and most intimate connexion between the state and development of the brain, and the exercise and power of the moral and mental phenomena of our being; but it is, perhaps, even more than doubtful whether there be so strict a localization —to adopt a Broussaian term—of the different attributes of the mind, as has been so ingeniously maintained by Gall and his disciples.

We are quite willing, however, to admit that the labours of the phrenologists have been attended with most marked and unquestionable good in all subsequent metaphysical and ethical inquiries, by giving a character of precision and exactitude to the description of mental and moral phenomena, and to the intimate connexion between them and the condition of our corporeal frames, which had been far too much neglected by all former inquirers. So it has been with the Broussaian system, in reference to practical medicine. While we call in question the absolute truth of its doctrines, and object to many of the conclusions which have been urged upon us, we cannot fail to admit that it has achieved a great and permanent good by directing the minds of medical men to a more minute and searching examination of local and special changes than was ever done before.—*Gazette Medicale.*

